# A novel portable Raman scattering platform for antibiotic screening in pig urine

**DOI:** 10.14202/vetworld.2023.204-214

**Published:** 2023-01-29

**Authors:** Nungnit Wattanavichean, On-uma Nimittrakoolchai, Noppadon Nuntawong, Mati Horprathum, Pitak Eiamchai, Saksorn Limwichean, Pacharamon Somboonsaksri, Donruethai Sreta, Sirilak Meesuwan

**Affiliations:** 1 School of Materials Science and Innovation, Faculty of Science, Mahidol University, Phutthamonthon, Nakhon Pathom, Thailand; 2 SCI Innovatech Co., Ltd., Bangkhasor, Amphur Mueang, Nonthaburi, Thailand; 3 National Electronics and Computer Technology Center, National Science and Technology Development Agency, Khlong Luang, Pathum Thani, Thailand; 4 Faculty of Veterinary Medicine, Rajamangala University of Technology Tawan-ok, Sriracha, Chonburi, Thailand

**Keywords:** antibiotics, pig, Raman spectroscopy, urine

## Abstract

**Background and Aim:**

Public health and food safety are gaining attention globally. Consumer health can be protected from chemical residues in meat by early detection or screening for antibiotic residues before selling the meat commercially. However, conventional practices are normally applied after slaughtering, which leads to massive business losses. This study aimed to use portable surface-enhanced Raman spectroscopy (SERS) equipped with multivariate curve resolution-alternation least squares (MCR-ALS) to determine the concentrations of enrofloxacin, oxytetracycline, and neomycin concentrations. This approach can overcome the problems of business loss, costs, and time-consumption, and limit of detection (LOD).

**Materials and Methods:**

Aqueous solutions of three standard antibiotics (enrofloxacin, oxytetracycline, and neomycin) with different concentrations were prepared, and the LOD for each antibiotic solution was determined using SERS. Extracted pig urine was spiked with enrofloxacin at concentrations of 10, 20, 50, 100, and 10,000 ppm. These solutions were investigated using SERS and MCR-ALS analysis. Urine samples from pigs at 1 and 7 days after enrofloxacin administration were collected and investigated using SERS and MCR-ALS to differentiate the urinary enrofloxacin concentrations.

**Results:**

The LOD of enrofloxacin, oxytetracycline, and neomycin in aqueous solutions were 0.5, 2.0, and 100 ppm, respectively. Analysis of enrofloxacin spiking in pig urine samples demonstrated the different concentrations of enrofloxacin at 10, 20, 50, 100, and 10,000 ppm. The LOD of spiking enrofloxacin was 10 ppm, which was 10 times lower than the regulated value. This technique was validated for the first time using urine collected on days 1 and 7 after enrofloxacin administration. The results revealed a higher concentration of enrofloxacin on day 7 than on day 1 due to consecutive administrations. The observed concentration of enrofloxacin was closely correlated with its circulation time and metabolism in pigs.

**Conclusion:**

A combination of SERS sensing platform and MCR-ALS is a promising technique for on-farming screening. This platform can increase the efficiency of antibiotic detection in pig urine at lower costs and time. Expansion and fine adjustments of the Raman dataset may be required for individual farms to achieve higher sensitivity.

## Introduction

The swine industry is a major contributor to global livestock markets. Pigs are the most popular animals that are considered human primary protein sources. Their meat is the most widely consumed globally, especially in Europe and Southeast Asia, due to their price and palatability. According to the USDA, over 105 million metric tons of pork were consumed in 2021, which implies a requirement of more than 752 million pigs for the global consumer chain [1]. This number is predicted to increase by at least 13% next year. Since the swine industry governs a large portion of the total meat market, a series of laws and regulations have been launched to protect consumer health [2]. A concern is the level of antibiotic residues in pork. Antibiotics are extensively used at high dosage in swine farming to accelerate growth, reduce mortality, improve fertility, and prevent various breast, blood, respiratory, and gastrointestinal bacterial infection in pigs [3, 4]. Antibiotics are crucial in maintaining swine health during their growth and are part of general veterinary practices [5]. Unfortunately, excessive use of antibiotics can potentially result in antibiotic residues in human food; such residues are demonstrably a primary issue in human public health [4]. These residues are involved in various issues, such as antimicrobial resistance, cancer and allergy triggers, bone marrow toxicity, mutations, and reproductive disorders [4, 6]. The predominant antibiotics that are commonly exploited as veterinary drugs in pig farms are enrofloxacin, oxytetracycline, and neomycin because of their broad spectrum and growth-promotion effects [7]. Therefore, the concentrations of these antibiotics must be tested before the pork enters the human food supply chain.

To protect the consumers from chemical residues in pork, the traditional technique involves analyzing trace contamination with antibiotics directly from pork samples after they are slaughtered. This method causes major business losses if high concentrations of antibiotics are detected because the whole batch of pork cannot be sold. Consequently, antibiotic detection in pig urine has been developed to avoid such high costs [8, 9]. The common technique for urinary detection of antibiotics includes high-performance liquid chromatography or mass spectrometry, which provide very accurate results as well as include super-high sensitivity [7, 10, 11]. However, it is a time-consuming procedure and requires complicated sample pre-treatment and well-trained laboratory personnel. A cheaper detection method, such as the European Four Plate Test is a popular technique but requires at least 18 h for complete evaluation [12]. A newer generation of tests is based on colorimetric testing secondary to chemical interactions [13]. Although it is used in pig farms and provides high sensitivity and specificity, it requires 2.5–3.5 h in an incubator for the results. Since screening for antibiotic residues in pig urine must be performed before the pig reach the slaughterhouses, a rapid and on-site technique should be considered. Raman spectroscopy is a spectral technique based on the interaction between light and matter. It is used to identify the molecular vibrations of chemicals when triggered by excitation light. Raman spectroscopy is a non-destructive technique that does not require sample preparation. Therefore, it yields remarkable benefits as a detection tool, particularly for rapid and *in situ* observations. Unfortunately, the signal from this technique is relatively weak in biological samples. To identify trace elements, such as antibiotics in pig urine, the signals need to be amplified using surface-enhanced Raman spectroscopy (SERS). Surface-enhanced Raman spectroscopy enhances the local electric field of the substrate and, thus, enhances the signal. Surface-enhanced Raman spectroscopy has been widely used to detect various trace elements, such as pesticides and antibiotics in foods, agricultural products, and contaminated water [14–18]. The previous studies have proven that SERS can significantly improve the detection limit for many compounds [19–21]. However, studies are currently limited to human samples. Cephalosporins have been detected in human urine [19]. All determined concentrations were lower than the minimal requirements for therapeutic drug monitoring, which is as low as 7.5 μg/mL for ceftriaxone. Another study used SERS to detect sulfonamides in pig urine using gold nanoparticles as an enhancing substrate [20]. Pig urine spiked with ractopamine and identified at concentrations of 0.1–10 mg/mL [21]. This study mentioned the characteristic peaks of antibiotics that are suitable for quantitative analysis by coupling them with density functional theory calculations. However, the samples require extra steps of pre-treatment. To the best of our knowledge, no studies have used SERS to screen pig urine for enrofloxacin, oxytetracycline, or neomycin, which are antibiotics commonly used in the swine industry.

This study, aimed to investigate the concentrations of these crucial antibiotics in aqueous solutions and swine urine using SERS. A silver nanorod substrate was selected because of its high electric field enhancement [22]. The lowest detection value for each antibiotic in an aqueous solution was determined. Further analyses used multivariate curve resolution-alternating least squares (MCR-ALS) to separate the trace antibiotic concentrations from other urine components [23]. The characteristic Raman spectrum of each antibiotic in the urine was clearly observed and the relationship between the Raman signal and antibiotic concentrations was determined.

## Materials and Methods

### Ethical approval

The research protocols were approved by the Animal Ethic Committee of Rajamangala University of Technology Tawan-ok (Ref. No. RUMTTO-ACUC-2-2021-008) as per the guidelines for the Care and Use of Experimental Animals of the National Research Council of Thailand.

### Study period and location

The study was conducted from January 2021 to June 2022. The research was conducted at Laboratory of Clinical Pathology, Faculty of Veterinary Medicine Rajamangala University of Technology Tawan-ok and Opto-Electrochemical Sensing Research Team, Spectroscopic and Sensing Devices Research Group, National Electronics and Computer Technology Center.

### Experimental design

Twenty piglets were examined after weaning (males; age, 28 days) by the Faculty of Agriculture and Natural Resource Farm, Rajamangala University of Technology Tawan-ok. These healthy pigs had an average weight of 7 kg and no history of antibiotic treatment. Twenty piglets were divided into four groups (5 pigs/group) and were either untreated or treated with enrofloxacin, oxytetracycline, or neomycin. Enrofloxacin was administered subcutaneously at a rate of 5 mg/kg body weight/day for three consecutive days. Urine samples were collected from all pigs before administering the drug on day 1 and after the last administration on day 7. The samples were stored at −20°C for further analysis. We collected 1.5 mL of urine from each pig in a sterile tube and centrifuged at 4500× *g* for 10 min. The supernatant was transferred to a clean polypropylene tube, zeolite was added, and the mixture was kept aside for 2 h. The solution was filtered using a 0.2 μm filter paper (Whatman International Ltd., England) for Raman spectroscopy.

### The antimicrobial residue screening test kit (CM-test)

The urine samples were tested for antimicrobials (enrofloxacin, oxytetracycline, and neomycin) residues using an antimicrobial residue screening test kit (Determination of Drug Residue in Meat Test Kit, Asianmedic Co., Ltd., Thailand) [13].

### Antibiotic detection

Antibiotic detection was performed using SERS (ONSPEC, SCI Innovatech Co., Ltd., Thailand) using silver nanorods as the substrate. The substrate was prepared using a direct current magnetron sputtering system combined with a glancing-angle deposition technique [22]. Raman measurements were performed using a handheld Raman spectrometer (Mira M-3, Metrohm AG, Switzerland) with a 785-nm excitation laser. The laser power was 5 mW for measurements in both the solutions and on SERS substrate. The accumulation time for Raman measurements from the solution and on SERS substrate was 20 and 5 s, respectively. Three standard antibiotics (enrofloxacin [100 mg/mL; Bezter, Enro tec 500, Betagro Public Co., Ltd., Thailand], oxytetracycline [50 mg/mL; Oxyclin, General Drugs House Co., Ltd., Thailand], and neomycin [500 g/kg; Neomycin-500, Neotech Impex Co., Ltd., Thailand]) were procured from a veterinary pharmacy and used without further purification.

### Antibiotic spectra and limit of detection (LOD) in aqueous solution

Standard antibiotics were used to verify the Raman characteristic peaks of each antibiotic and for further analysis using MCR-ALS. The standard Raman spectrum of the antibiotics was recorded in a solution using a glass vial and on the SERS substrate. The sample was prepared by dropping 5 μL on SERS substrate and drying it for 5 min. Three Raman spectra were directly assessed per sample. After obtaining the standard spectra of all antibiotics, LOD was determined to observe the lowest concentration of detection using only the SERS substrate. Standard solutions of antibiotics were prepared at different concentrations (0.01, 0.1, 0.5, 1, 2, 5, 10, 20, 50, and 100 ppm) in distilled water. This range of concentrations covers the minimum concentration allowed by the regulations. The Raman spectrum of each antibiotic was recorded until LOD was determined.

### Raman spectra of antibiotics in pig urine

Raman spectra of urine collected from pigs who did not receive antibiotics were obtained for negative control. The control urine was spiked with antibiotics at various concentrations (10, 20, 50, 100, and 10,000 ppm) and was used as references. After administering enrofloxacin, pig urine was collected on days 1 and 7 for on-farming antibiotic determination. Subsequently, 5 μL of each urine sample was dropped on the SERS substrate and dried. Surface-enhanced Raman spectroscopy measurements were collected randomly from the substrate surface with at least 10 spectra for each concentration. All Raman spectra of enrofloxacin were pooled for further analysis using MCR-ALS.

### Spectral analysis

Multivariate curve resolution-alternating least squares is a multivariate spectral analysis method. This is a method of mathematical approximation from two non-negative matrices. Therefore, this method is used to extract a few principal spectral components from complex information obtained from numerous Raman spectra without prior spectral information. Multivariate curve resolution-ALS was performed using the IGOR Pro (WaveMatrics, Inc., Oregon, USA). More than 300 spectra from three datasets of enrofloxacin in water, spiked pig urine, and urine samples were analyzed using MCR-ALS to verify the concentration of antibiotics [24]. Standard antibiotics Raman spectra were used as references for the analysis.

## Results

### Raman spectra of standard antibiotics

**Figure-1:**
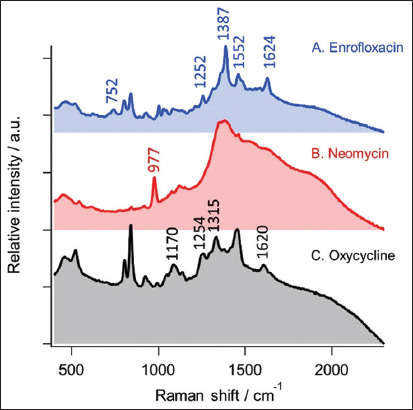
Raman spectra of standard antibiotics (A) enrofloxacin, (B) neomycin, and (C) oxytetracycline.

The Raman spectra of three standard antibiotics (enrofloxacin, neomycin, and oxytetracycline) in water are shown in Figure-1. The dominant peaks in the Raman spectrum of enrofloxacin include 752, 1252, 1387, 1552, and 1624/cm [25] (Figure-1A). Figure-1B shows the Raman spectrum of neomycin with a dominant peak at 977/cm [26]. The characteristic Raman peaks at 1170, 1254, 1315, and 1620/cm [27] of oxytetracycline are shown in Figure-1C. Signals at 803 and 835/cm were noted for both enrofloxacin and oxytetracycline.

### Raman signal enhancement

**Figure-2:**
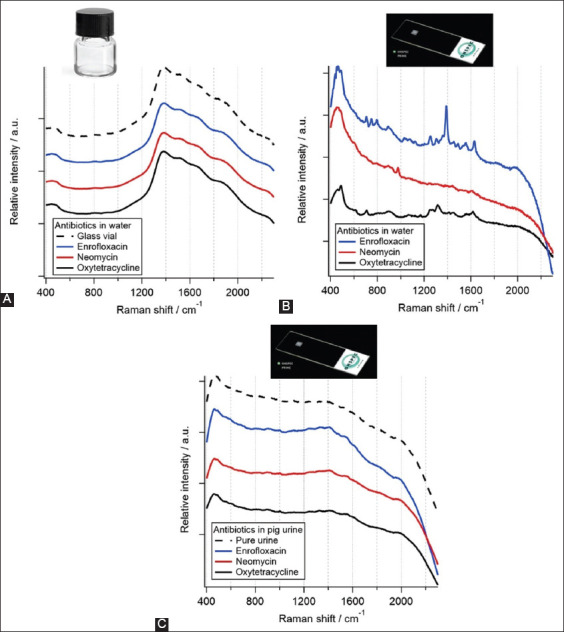
Raman spectrum comparisons of standard antibiotics at a concentration of 100 ppm (A) in water without surface-enhanced Raman spectroscopy (SERS), (B) in water with SERS, and (C) in urine with SERS.

To verify the detection of trace antibiotics, 100 ppm solution each of enrofloxacin, neomycin, and oxytetracycline was prepared in water. The Raman spectra of the antibiotics were measured as a bulk solution in a glass vial as well as on the SERS substrate. None of the antibiotics were identified at a concentration of 100 ppm (Figure-2A). The only signal observed was that from the glass vial (dashed line) because the concentration of the antibiotics was too low. The same concentrations of antibiotics were detected on the SERS substrates. The Raman signal showed a dramatic enhancement for every antibiotic on the SERS substrates (Figure-2B). The results confirmed the successful enhancement of the Raman signal for the detection of trace elements on the SERS substrate. The detection was further challenged by spiking 100 ppm of antibiotics in pig urine. These samples were detected using SERS to determine the effect of pig urine on the Raman signal. The Raman spectra of 100 ppm antibiotics in pig urine are shown in Figure-2C. Although Raman signals were noted in water, they were very difficult to identify in pig urine. This result was affected by the fact that urine contains an abundance of organic compounds. Most organic molecules demonstrated very strong auto-fluorescent signals, which were broad with high intensities. Therefore, they masked the other signals of interest.

### Limit of detection determination

**Figure-3:**
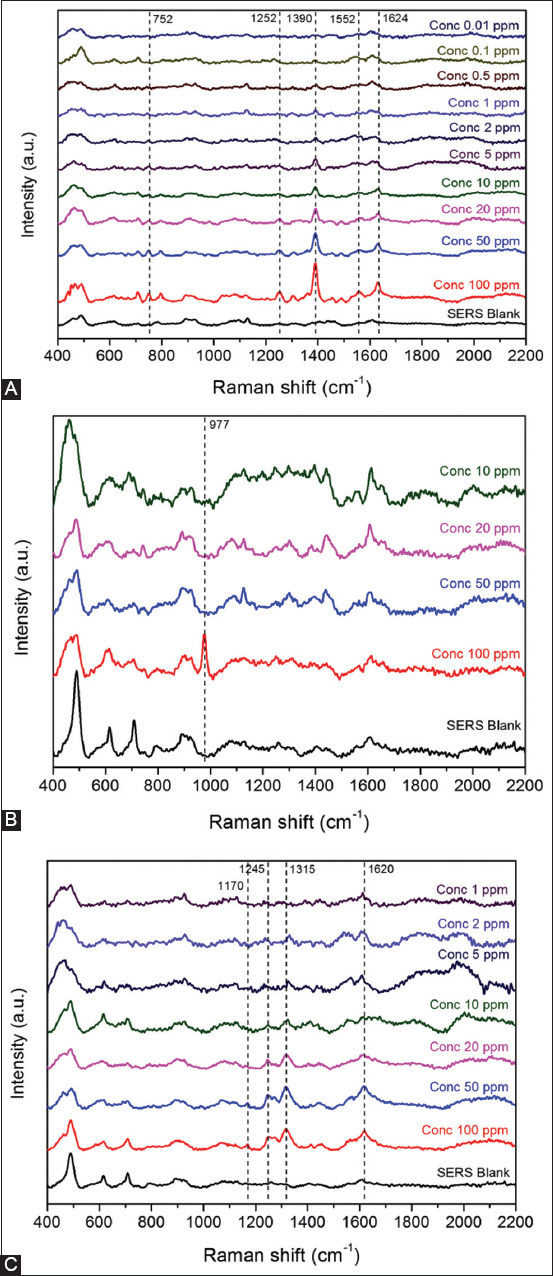
Limit of detection determination of (A) enrofloxacin, (B) neomycin, and (C) oxytetracycline solution at different concentrations.

Limit of detection of each antibiotic was observed in water and baseline subtraction was performed. As the antibiotic concentration decreased, the Raman signal decreased as well. The LOD was defined as the lowest concentration at which the characteristic peak of each antibiotic could still be identified. Figure-3 shows the average Raman spectra for various antibiotic concentrations. Although the Raman spectra were obtained at different locations on the SERS substrate, all three spectra demonstrated almost identical peak intensities for each concentration, which confirms the repeatability of the SERS substrate. Peaks at 1390 and 1624/cm were used to identify enrofloxacin, whereas neomycin was identified by a signal at 977/cm, and oxytetracycline was identified by peaks at 1315 and 1620/cm. The LOD of the same antibiotics on SERS were 0.5, 2.0, and 100 ppm (Table-1) [2]. The dominant Raman peaks of each antibiotic could still be amplified until the antibiotic solution was diluted to a concentration that could not be enhanced by SERS, thus resulting in the disappearance of the Raman peaks. As can be seen by the results in Figure-3, the LODs for detecting these antibiotics on the substrate in aqueous solutions were 0.5, 2.0, and 100 ppm.

**Table-1:** The LOD on SERS substrates for antibiotics detection in this study and the MRL in regulation [2].

Antibiotic drug	LOD in this study (ppm or µg/mL)	MRL [2] (ppm or µg/mL)
Enrofloxacin	0.5	100
Oxytetracycline	2.0	100
Neomycin	100	100

LOD=Limit of detection, SERS=Surface-enhanced Raman spectroscopy, MRL=Maximum residue limit

### Multivariate analysis with MCR-ALS

**Figure-4:**
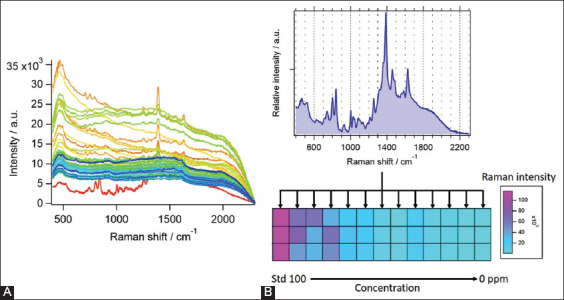
Raman spectra of enrofloxacin in water measured on surface-enhanced Raman spectroscopy substrate. (A) A total of 59 spectra of enrofloxacin in different concentrations and (B) Raman spectrum of the separated component from enrofloxacin (top) and its intensity (bottom).

Enrofloxacin was selected for further analysis using MCR-ALS because it demonstrated a strong Raman signal. Multivariate analysis was used to separate the antibiotic signals from those of other ingredients in pig urine. After mixing enrofloxacin with pig urine extract, the dominant peak of enrofloxacin could not be observed at the low concentrations. Conversely, some peaks were observed only at the high concentrations due to the interference of the fluorescence signal from the urine, which lowered the enhancement of the antibiotic signal on SERS. The interference of the fluorescence signal from the urine could be solved using MCR-ALS, which could differentiate the numerous mixed Raman spectra of enrofloxacin in extracted pig urine into a single component. The method was primarily verified using enrofloxacin in water on a SERS substrate. The Raman spectra were recorded at enrofloxacin concentrations of 0 (negative control), 0.01, 0.1, 0.5, 1, 2, 5, 10, 20, 50, and 100 ppm. The Raman spectra of the enrofloxacin standard were also included. The total number of Raman spectra obtained in this study was 59. Figure-4A shows all the spectra obtained for enrofloxacin in water. The Raman signal varied from obvious to barely visible depending on the concentration of the drug. The spectra were analyzed using a six-component model to separate the background noise, fluorescence, and signal from enrofloxacin. Subsequently, the signal from enrofloxacin was excluded from other background noise and fluorescence. The separated Raman spectrum of enrofloxacin is shown in Figure-4B. In addition, the Raman intensities of the extracted component depend on the concentration of enrofloxacin in each sample. The image of Raman intensity is shown in Figure-4B (below) with the color scale on the right. The concentration of enrofloxacin is shown below Raman image for comparison. The LOD for enrofloxacin detection was reduced from 0.5 ppm to 0.1 ppm after the analysis, which enhanced the detection limit.

**Figure-5:**
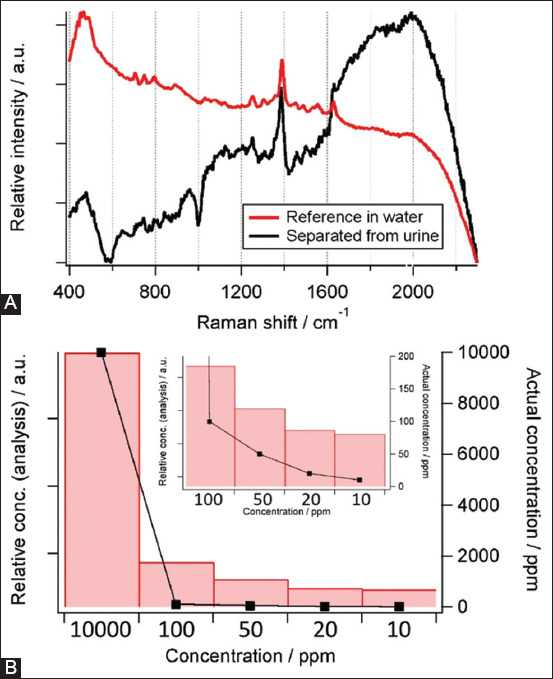
(A) Separated enrofloxacin component from multivariate analysis (black) comparing to reference enrofloxacin spectrum (red) (B) Concentration comparison between Raman signal and actual enrofloxacin concentration at 10, 20, 50, 100 and 10,000 ppm, extracted using multivariate curve resolution-alternation least squares. The inlet shows a magnified image of 10, 20, 50, and 100 ppm conditions.

A similar analysis was performed for enrofloxacin in the pig urine samples. Pig urine was spiked with enrofloxacin at concentrations of 10, 20, 50, 100, and 10,000 ppm. At the low concentrations (10−100 ppm), the dominant peak of enrofloxacin at 1390 and 1624/cm could not be observed (Figure-2C). However, at the high concentration (10,000 ppm), the characteristic peaks of enrofloxacin were slightly observed. To identify trace concentrations of enrofloxacin, all the Raman spectra at different concentrations of enrofloxacin were pooled. The spectra from 10, 20, 50, 100, and 10,000 ppm of enrofloxacin in pig urine were used, which resulted in a total number of 250 spectra for MCR-ALS. The raw spectra revealed an almost flat signal with a very strong fluorescent background. Six-principal components were extracted from the analysis. The components of organic fluorescence and background noise were excluded from the study. The component that belonged to enrofloxacin is shown in Figure-5A (black spectrum). The extracted component was confirmed to be enrofloxacin based on a reference spectrum of enrofloxacin (red spectrum). The intensity of this Raman component was related to the actual spiking concentration of enrofloxacin (Figure-5B). The Raman intensity of the separated component is indicated by the red bar, whereas the actual spiking concentration of enrofloxacin is shown by black line. The spiking concentrations of enrofloxacin were 10, 20, 50, 100, and 10,000 ppm, as indicated on the horizontal axis. A trend of decreasing Raman intensity along with the actual enrofloxacin concentration was clearly observed. The inlet in Figure-5B highlights a strong correlation between the enrofloxacin concentration and the Raman signal at very low concentrations of 10–100 ppm. The Raman intensity was sensitive to a very low concentration of antibiotics.

**Figure-6:**
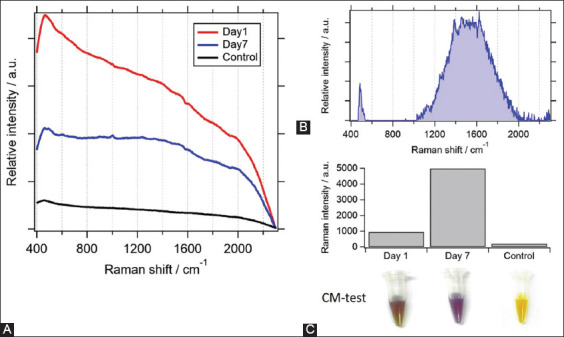
(A) Raman spectra of collected pig urine (black- control, red- day 1 urine, and blue- day 7 urine). (B) Extracted Raman spectrum of enrofloxacin using multivariate curve resolution-alternation least squares analysis (C) Raman intensity on urine samples of on days 1 and 7, in comparison with control urine without enrofloxacin injection (right). Color development on CM-test in day 1, day 7, and control samples (below).

Determination of enrofloxacin from subcutaneous injections was confirmed via multivariate analysis. Enrofloxacin was injected into the pigs at a concentration of 50 mg/kg body weight on days 1, 2, and 3. Urine samples were collected on days 1 and 7 to measure the enrofloxacin concentration. Control samples were collected from pigs who did not receive any antibiotics. The original Raman spectra of the pig urine are shown in Figure-6A. The Raman spectrum from the control condition appeared as an almost flat background. Urine samples from days 1 and 7 showed similar signals. However, a higher signal at 1000/cm was observed in the urine of day 7. All 53 Raman spectra were pooled and multivariate analysis was performed. The four-component model was applied for spectral separation. According to the MCR-ALS analysis, characteristic peaks around 1400 and 1600/cm of the enrofloxacin spectrum were identified (Figure-6B). Urine samples from day 7 revealed a higher Raman intensity of enrofloxacin than those of the samples from day 1 (Figure-6C). No signal was observed for enrofloxacin in the negative control. The same batch of urine was cross-checked using CM-test, which is the standard screening test kit used to detect antimicrobial residues in meat, serum, and urine based on bacterial inhibition in appropriated bacterial culture media tubes. The bacteria could grow and produce acids in the samples without antibiotics, which resulted in a color change from yellow to purple. Therefore, the absence and presence of antibiotics in the sample were verified based on the yellow and purple color, respectively. According to Figure-6C, samples of day 7 developed a deeper purple color after CM-test than the samples of day 1. Therefore, the higher antibiotic concentration in the urine of day 7 urine was verified. However, on day 1, a slight color change was observed compared to day 0 (negative control).

## Discussion

The dominant peaks in the Raman spectrum of enrofloxacin at 752, 1252, 1390, 1552, and 1624/cm arise from the following vibration modes of enrofloxacin: Symmetric O-C-O stretching vibrations (1390/cm), benzene ring vibrations (1252/cm), and C=O stretching vibrations (1624/cm), methylene rocking modes (752/cm), and benzene ring stretching (1552/cm) [28]. The characteristic peaks at 1170, 1315, and 1620/cm of oxytetracycline was ascribed to C–C=O deformation, aromatic ring in-plane H bending, amine C–N stretching, and aromatic ring stretching, respectively [29]. For neomycin, only one peak at 977/cm was attributed to CH_2_ rocking and C-C stretching [26, 30].

### Raman enhancement with SERS substrate

The Raman spectra of antibiotics obtained using the standard solution technique and the SERS substrate have been reported. Our results demonstrate that the nanorod microstructure on the SERS substrate can greatly enhance the SERS signal, which makes Raman spectroscopy a promising technique for detecting trace elements, especially for antibiotic determination. At the same antibiotic concentration of 100 ppm, the characteristic signal of the antibiotic in the solution could not be observed. Only the signal from the glass vial was noted. However, the signal significantly increased on using a SERS substrate. Several characteristic peaks indicated the presence of antibiotics even at very low concentrations. This was due to the surface plasmon resonance effect, which creates a hotspot for detection [31]. In this region, the electromagnetic signal, including the Raman effect, is highly gained. This result corresponds with those of other studies as silver nanostructures are demonstrably well enhanced for Raman measurements and have been used in many applications [32–34]. In addition, the size of the silver nanostructure also affects the magnitude of the signal enhancement [35]. Kao *et al*. [36] used a combination of Ag nanocubes and Ag octahedra to increase the Raman signals of metabolites in human urine. They found that the signal could be enhanced up to 10^12^-fold. Another experiment provided large-scale self-assembly of gold nanoparticle arrays to identify various drugs in urine [37]. Methamphetamine was used at a concentration of 1 ppm. Surface-enhanced Raman spectroscopy also works well for the detection of chronic kidney disease when coupled with principal component analysis and linear discriminant analysis [38]. The experiment with swine urine was also successfully conducted with SERS to detect β-agonists and ractopamine at the lowest concentration of 2 and 0.1 μg/mL, respectively [39].

### Limit of detection determination of antibiotics

The lowest detection limit is crucial in the development of any type of sensor. In our study, the LOD of enrofloxacin could go down to 0.5 ppm as a solution in water. This value can be validated with the naked eye as well as using multivariate analysis (MCR-ALS) from all three samples at 0.5 ppm, which confirms the repeatability of this technique. According to the regulations, enrofloxacin concentration in swine urine should be lower than 100 ppm [2]. Therefore, the classification of ready-to-slaughter pigs can be confirmed using our method. In antibiotic detection, some researchers have attempted to use SERS to detect insoluble enrofloxacin in water [25]. However, for soluble enrofloxacin, only the limit of the quantification has been published on the ppm scale [40]. Therefore, our work is the first report of the detection of enrofloxacin at a very low concentration of 0.5 ppm. Detection is rarely seen in swine urine samples. Only the determination of β-agonists, ractopamine, and sulfonamides, which are the older generation of antibiotics, has been reported previously [20, 21, 39]. Although enrofloxacin is another widely used antibiotic in swine feedstock, there are limited reports on the residues of this antibiotic. Therefore, this study focused on its concentration in the collectible urine sample. Sampling pig urine allows farmers to indirectly check antibiotic levels before sending them to slaughterhouses. The concentration of antibiotics in pig urine is related to that in their meat [11]. Therefore, farmers can avoid wasting their products due to the over-concentration of antibiotics than that allowed by regulations. The concentration of enrofloxacin in pig urine was determined in this study. The main obstacle for detecting antibiotics (such as enrofloxacin) in pig urine is the fluorescent background [41, 42]. Urine contains various organic substances, such as zearalenone, zeranol, taleranol, and zearalenol [43]. These organic compounds consist of aromatic ring structures as well as complex macromolecules, which eventually contribute to a large fluorescent background in Raman measurements [44]. As demonstrated in our results, the signal from antibiotics almost vanished due to the broad fluorescent background in all urine samples. In this case, multivariate analysis has been utilized to extract only pure signals from enrofloxacin by separating the principal components that may be present in the Raman spectra. The analysis revealed that pig urine spiked with enrofloxacin contained signals from the SERS substrate background, multiple auto-fluorescent backgrounds, and characteristic peaks of enrofloxacin. Only the pure signal of enrofloxacin was selected for further analysis at various concentrations. Our results revealed that the concentration of enrofloxacin in pig urine can be identified down to a scale of 10 ppm, which is 10 times lower than that of required by regulations [2]. This method was reproducible in all five samples at the same concentration. This classification can help in screening for pigs on livestock farms that are ready for slaughterhouse. Furthermore, the classification has a higher sensitivity for lower concentrations (below 100 ppm), which allows this classification for further applications that require very high sensitivity. An increasing number of Raman spectra from the samples can be obtained to improve the sensitivity of the method in the future.

### Enrofloxacin determination in the collected pig urine

Enrofloxacin is an antibiotic used in the preparation of quinolone carboxylic acid derivatives. It is the most effective against Gram-negative bacteria that affect the respiratory, gastrointestinal, and urinary tracts of cattle, pigs, and poultry. Enrofloxacin is also one of the most commonly used antibiotics on pig farms. In pigs, enrofloxacin has been used for the treatment and control of swine respiratory diseases, including those caused by *Actinobacillus pleuropneumoniae, Pasteurella multocida, Haemophilus parasuis*, *Streptococcus suis, Bordetella bronchiseptica*, and *Mycoplasma hyopneumoniae* as well as in the control of *Escherichia coli* Post-weaning diarrhea [45]. Antimicrobial resistance can develop during the treatment of bacterial infections in pig farms. To the best of our knowledge, this study was the first to demonstrate the concentration of enrofloxacin in pig urine. Pigs were injected with enrofloxacin at 50 mg/kg body weight on days 1, 2, and 3. Urine samples were collected on days 1 and 7 for Raman measurements. The original Raman spectra from pig urine demonstrated a high fluorescence peak and the random background characteristics varied between samples. Only a few spectra revealed small characteristic peaks of enrofloxacin due to enhancement by the SERS substrate. Multivariate analysis was performed to extract better information from enrofloxacin. This result confirmed a higher urinary concentration of enrofloxacin on day 7 than that on day 1. Control pig urine was also used to validate this technique. No enrofloxacin signal was detected in the control sample. The increase in urinary enrofloxacin concentration was due to consecutive injections on days 1−3. On the first day of injection, enrofloxacin mostly enters the bloodstream and is metabolized into ciprofloxacin through the hepatic system [46] before being distributed throughout the pig body; however, 80% of fluoroquinolones are excreted via the urine by the renal system [47]. Subsequently, the enrofloxacin concentration reached a level that could reduce bacterial infection on day 3 [48]. Sequentially injected enrofloxacin on days 2−3 causes high concentrations of antibiotics in bloodstream as well as in the excretory system. Kim *et al*. [49] found that cabadox concentration was the highest on day 5 in the serum and colon microbiota. Another experiment conducted with manure samples confirmed that the maximum excretion of difloxacin and sarafloxacin occurred on days 4−6 after oral administration [50]. The maximum urinary antibiotic concentration was found to be on day 5. As the half-life of enrofloxacin in urine is 1.48 days, it is demonstrably retained in urine for approximately 3 weeks after application [51]. This phenomenon was also observed in our results, even though the Raman spectra were obtained from 10 pigs. Therefore, our Raman platform can provide a reproducible result within our batch as well as other studies. Furthermore, the detection of antibiotics in urine is better than that in muscles since antibiotics are frequently detected and present at higher concentrations in urine [11]. Therefore, urine may be a promising sample for on-farm antibiotic detection. Detecting urinary antibiotics not only prevent business losses but also improves detection accuracy. Our demonstrated SERS measurements highlight the possibility of on-farming detection in the future. The detection was proved to be 10 times lower than the regulated limit and could differentiate the concentration of antibiotics from the collected pig urine.

## Conclusion

Surface-enhanced Raman spectroscopy coupled with MCR-ALS was used for on-farming antibiotic testing of pig urine. This procedure provides primary screening for antibiotics in pigs before delivery to slaughterhouses. Our results revealed LOD of 0.5, 2.0, and 100 ppm for enrofloxacin, oxytetracycline, and neomycin detection in water. The LOD can be as low as 10 ppm for pig urine in the enrofloxacin-spiked sample, which is 10 times lower than the European regulation. Finally, to the best of our knowledge, this study was the first to monitor the actual urine collected from pigs 1 and 7 days after enrofloxacin injection. The results demonstrated a relatively high enrofloxacin concentration in the urine on day 7 compared to that on day 1. This result was related to the higher accumulation of enrofloxacin in urine due to consecutive injections. Furthermore, the urinary excretion of enrofloxacin corresponds to the circulation lifetime and metabolism of the drug in pigs. The SERS sensing platform combined with MCR-ALS can increase the efficiency of antibiotic detection in pig urine by differentiating mixed Raman spectra, which promotes higher precision. Therefore, this sensing procedure can overcome the detection limit of on-site and rapid antibiotic screening. The urinary Raman spectrum has significant variations according to pig’s metabolism (urea and ammonia content); therefore, increasing the Raman spectra for analysis can enhance the sensitivity of this technique. In reality, this platform requires fine adjustments for a specific farm, which depends on the pig feed, antibiotic treatment, age, and sex. In the future, it may be applied to other toxic chemical residues in the swine industry.

## Authors’ Contributions

NW, ON, NN, MH, PE, SL, PS, DS, and SM: Conceived and designed the experiment protocols. SM and DS: Performed animal experiments. NW, ON, NN, MH, PE, SL, and PS: Performed experiment and analyzed data of Raman Spectroscopy test. NW, ON, NN, MH, PE, SL, PS, DS, and SM: Created the paper. All authors have read and approved the final manuscript.
